# cAMP response element‐binding protein and Yes‐associated protein form a feedback loop that promotes neurite outgrowth

**DOI:** 10.1111/jcmm.13324

**Published:** 2017-08-31

**Authors:** Lei Chen, Peimin Feng, Anjiao Peng, Xiangmiao Qiu, Xi Zhu, Shixu He, Dong Zhou

**Affiliations:** ^1^ Department of Neurology West China Hospital Sichuan University Chengdu Sichuan China; ^2^ Department of integrated traditional and western medicine Hospital of Chengdu University of Traditional Chinese Medicine Chengdu Sichuan China

**Keywords:** cAMP response element‐binding, Yes‐associated protein, neurite outgrowth

## Abstract

The cAMP response element‐binding (CREB) protein is a member of the CREB/activating transcription factor family that is activated by various extracellular stimuli. It has been shown that CREB‐dependent transcription stimulation plays a key role in neuronal differentiation and plasticity, but the underlying mechanisms remain largely elusive. Here, we show that Yes‐associated protein (YAP) is a direct target induced by CREB upon retinoic acid (RA)‐induced neurite outgrowth stimuli in N2a cells. Interestingly, YAP knockout using the CRISPR/Cas9 system inhibits neuronal differentiation and reduced neurite length. We further show that YAP could directly bind to CREB *via* its N‐terminal region, and loss of YAP results in instability of phosphorylated CREB upon neurite outgrowth stimuli. Transient expression of YAP could largely restore CREB expression and neurite outgrowth in YAP knockout cells. Together, our results suggest that CREB and YAP form a positive feedback loop that is critical to maintain the stability of phosphorylated CREB and promote neurite outgrowth.

## Introduction

Neurite outgrowth is a prerequisite step for neuronal differentiation and regeneration. Defects in neurite outgrowth might lead to abnormal synaptic polarization and plasticity, impaired axons and dendrites connectivity, and a wide range of central nervous system diseases and neurodegenerative disorders such as Alzheimer's disease and Parkinson's disease. Previous studies have shown that cAMP response element‐binding protein (CREB) plays a critical role in neuronal differentiation processes 1, 2, 3, 4, 5, 6. CREB is a ubiquitous transcription factor with particularly high expression in neuronal cells. Upon signals that induces neurite outgrowth, CREB is activated by ser133 phosphorylation and immediately induces expression of numerous target proteins containing cAMP response elements (CREs) in their promoters. For example, it has been shown that CREB plays a critical role in regulating NGF‐induced Bcl‐2 expression and promotes sympathetic neuron survival 7. Previous studies also showed that CREB controls expression of c‐fos, which is implicated in neuronal activation and injury repair 8. CREB could also modulate expression of regeneration‐associated genes (RAGs) to enhance axonal regeneration 9.

Accumulating evidence suggests a critical role of the Hippo pathway member YAP in regulating neuronal proliferation and differentiation. It has been shown that YAP1 is overexpressed in medulloblastomas and promotes neural precursor proliferation 10. Moreover, it has been shown that inhibition of Rho/Rho‐kinase (ROCK) pathway enhances neurite outgrowth in neural stem cells by up‐regulating YAP expression and activation of the Hippo pathway 11. YAP also interacts with the RA induced signalling to mediate early neural crest differentiation and migration 12. Notably, a recent study in liver cancer cells suggested that CREB enhances expression of YAP which in turn stabilizes CREB and promoted tumorigenesis 13. Given the essential roles of CREB and YAP in neurite differentiation, this raises the possibility that a mutual interaction between CREB and YAP might likewise occur in neuronal cells and is essential to neurite outgrowth.

To address the roles of CREB and YAP in promoting neurite outgrowth, we used RA‐induced N2a cell neurite outgrowth as a model system 14, 15. RA signalling has been shown to induce neurite outgrowth in various primary neuronal cultures and neuronal cell lines *via* inducing CREB phosphorylation and activation. Consistent with prior studies in liver cancer cells, our results suggest that YAP expression is tightly controlled by CREB in N2a cells during neurite outgrowth. By establishing YAP knockout cell lines using the CRISPR/cas9 system, we show that YAP is required for neuronal cell differentiation and neurite outgrowth. Interestingly, YAP interacts with CREB *via* its N‐terminal domain and prevents degradation of phosphorylated CREB upon neurite outgrowth stimuli. Our results revealed a positive feedback loop between CREB and YAP, which may be fundamental to neurite outgrowth and the development of neuronal circuits.

## Materials and methods

### N2a cell culture and treatment

N2a cells were cultured in 24‐well plates with DMEM (Thermo Fisher, Chelmsford, MA, USA) containing penicillin/streptomycin (Thermo Fisher) and 10% heat‐inactivated FCS (Thermo Fisher). To induce neurite outgrowth, N2a cells were treated with DMEM containing 0.1% serum and 5 μM RA (Sigma‐Aldrich, St. Louis, MO, USA) for 24 hrs. To inhibit CREB activity, cells were treated with 20 μM H89 (Cayman Chemical Company, Ann Arbor, MI, USA). To inhibit protein degradation, cells were treated with 50 μM MG132 (Cayman Chemical Company).

### Measurement of neurite outgrowth

Following RA treatment, differentiated N2a cells were stained with Neurite Outgrowth Staining Kit (Thermo Fisher) and imagined using an inverted microscope (Eclipse Ti‐E Inverted Microscope, Nikon, Japan). For neurite‐bearing cells, neurite length was defined as the distance between the centre of the cell soma and the tip of its longest neurite. For each treatment group, about 100 randomly selected cells were counted to obtain the proportion of neurite‐bearing cells and the average lengths of neurite using ImageJ software.

### CRISPR/Cas9 genome editing

CRISPR/Cas9 sgRNA targeting YAP was designed using CHOPCHOP online tool (http://chopchop.cbu.uib.no/): TGCCTCAGACCGTGCCCATG. The sgRNA was cloned into the pSpCas9(BB)‐2A‐GFP and transfected into N2a cells using Lipofectamine 2000 as per manufacturer's instructions (Thermo Fisher). GFP‐positive single cells were sorted out by flow cytometer and cultured into single colonies in 96‐well plates. The following sequencing primers were used to amplify the genomic region containing the putative mutations: YAP_F: 5′‐GACTCGGAGACCGACTTGG‐3′; YAP_R: 5′‐ATACCCTTACCTGTCGCGAGT‐3′.

### Western blot and real‐time PCR

Twenty micrograms of total cell lysates was separated on 4–12% SDS–PAGE (Thermo Fisher) and transferred onto a polyvinylidene difluoride (PVDF) membrane (Millipore, Bedford, MA, USA). After blocking with 10% of milk for 1 hr at room temperature, the membrane was subjected to immunoblotting using CREB (1:1000, Cell signaling, Danvers, MA, USA), p‐CREB (1:1000, Cell signaling), YAP (1:1000, Santa Cruz, Santa Cruz, CA, USA), tubulin (1:5000, Sigma‐Aldrich) and GAPDH (1:5000, Santa Cruz). Total RNA was purified using RNeasy Mini Kit (Qiagen, Hilden, Germany) and reverse‐transcribed into cDNA using M‐MLV reverse transcriptase (Promega, Madison, WI, USA). Real‐time PCR was performed with SYBR^®^ Green Quantitative RT‐qPCR Kit as per manufacturer's instructions using the following primers: CREB_F: 5′‐CATTAACCATGCCCAATGCAG‐3′; CREB_F: 5′‐ATGTGCGAATCTGGTATGTTT‐3′; GSK3A_F: 5′‐CAATATTGCAGTGGTC

CAGC‐3′; GSK3A_R: 5′‐ GGGAACTAGTCGCCATCAAG ‐3′; GSK3B_F: 5′‐CAATATT

GCAGTGGTCCAGC‐3′; and GSK3B_R: 5′‐ GGGAACTAGTCGCCATCAAG ‐3′.

### Proximity ligation assays (PLA)

Proximity ligation assays (PLA) assay was performed with the mouse/rabbit red starter Duolink kit (Sigma‐Aldrich) as per manufacturer's instructions. Briefly, N2a cells were fixed with 4% PFA for 15 min. and permeabilized with 0.5% Triton PBS for 15 min. Cells were incubated in the blocking buffer at 37°C for 1 hr in a humidified chamber. Next, cells were incubated with the mouse anti‐YAP Antibody (1:100, Santa Cruz) and the rabbit anti‐CREB (1:100, Cell Signaling) at 4°C overnight. After wash, cells were then incubated with the PLA probes at 37°C for 1 hr, and then ligation was performed at 37°C for 1 hr in a humidified chamber. Cells were then incubated with the amplification mix at 37°C for 2 hrs in a darkened humidified chamber. Cells were then mounted on coverslips using the mounting media supplied with the kit and imagined using an inverted microscope (Eclipse Ti‐E Inverted Microscope).

### Protein immunoprecipitation

N2a cells nuclear extract was prepared as previously described 16. Antibodies were cross‐linked to protein A beads in a 1:1 ratio using 6.5 mg/ml dimethyl pimelimidate (DMP) (Sigma‐Aldrich) and 20 μ of beads with antibody cross‐linked was added to nuclear extracts of cells from one 15 cm dish. After rotation at 4°C overnight, the beads were washed three times with 1 ml of 0.1% Triton X‐100 PBS buffer and then resuspended in 2× SDS buffer (0.35 M Tris‐Cl pH 6.8, 30% glycerol, 0.6 M DTT, 10% SDS, 0.0001% bromophenol blue) to elute the proteins. The elution was boiled for 10 min. before processed for Western blotting.

### Chromatin immunoprecipitation

Chromatin immunoprecipitation (ChIP) was performed with the Chromatin Immunoprecipitation (ChIP) Assay Kit (Millipore) as per manufacturer's instructions. Briefly, N2a cells induced by RA were treated with 10 μM forskolin for 45 min. at 37° and cross‐linked with 1.0% formaldehyde (Sigma‐Aldrich) for 10 min. at 22°, quenched with 0.125 M glycine for 10 min. Chromatin was sheared by sonication using Misonix Sonicator 3000 (Qsonica, LLC, Newtown, CT, USA) and processed for ChIP using protein A beads cross‐linked to anti‐CREB1 antibody and control IgG antibody. Isolated DNA was purified using a PCR Clean‐up kit (Qiagen) before amplification.

### Cell proliferation assay

Cells were cultured for up to 48 hrs to measure proliferation. For the indicated times, cells were incubated with MTT (0.5 mg/ml, Thermo Fisher) for 4 hrs at 37°C incubator and then treated with 100% DMSO. Viable cells were counted by reading the absorbance at 570 nm.

### Flow cytometry

Cells were seeded in six‐well plates for 24 hrs. Cells were then trypsinized and washed twice with PBS. Apoptosis was measured using the FITC Annexin V Apoptosis Detection Kit (Thermo Fisher) following the manufacturer's instruction. Cell populations were analysed by a FACSCalibur Flow Cytometer (BD, Chelmsford, MA, USA).

### Statistical analysis

Statistical analysis was also carried out using SPSS 16.0 software. Data were expressed as Mean ± S.E.M. of three biological replicates. Student's *t*‐test was used for comparisons between the groups, and *P*‐value < 0.05 was considered significant.

## Results

### CREB promotes YAP expression upon RA‐induced neurite outgrowth

**Figure 1:**
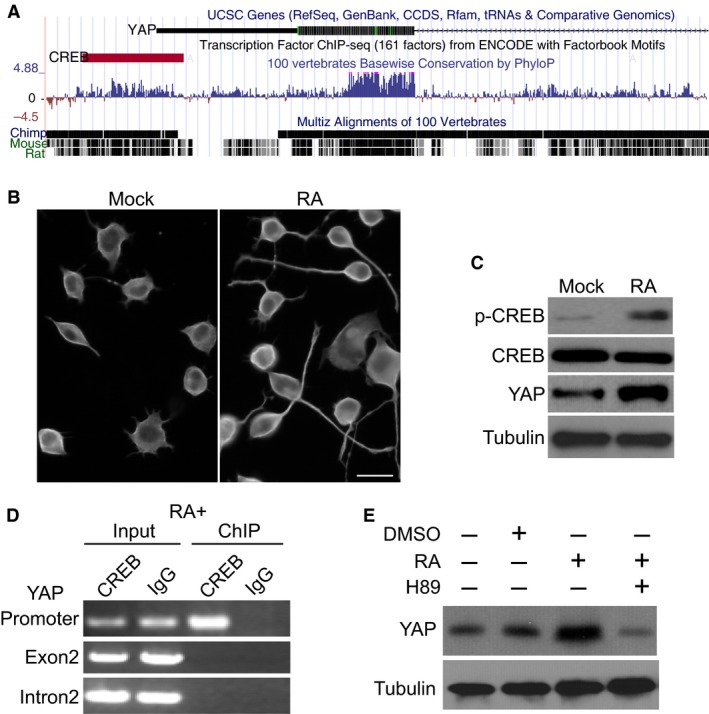
cAMP response element‐binding (CREB) promotes Yes‐associated protein (YAP) expression upon RA‐induced neurite outgrowth. (**A**) YAP gene and the CREB binding site are shown in UCSC genome browser. (**B**) Representative data of RA‐induced neurite outgrowth in N2a cells. (**C**) Western blotting of p‐CREB, total CREB, YAP and tubulin in cells treated with DMSO (Mock) or 5 μM RA for 24 hrs. (**D**) ChIP assay of CREB binding at YAP promoter or exon2 and intron2. (**E**) Western blotting of YAP expression in N2a cells treated with 20 μM CREB inhibitor H89.

After activation, CREB induces target gene expression by binding to the CRE motif 5′‐TGACGTCA‐3′ in the promoter region. Using ENCODE transcription factor chromatin immunoprecipitation sequencing (ChIP‐seq) data, we identified a putative CREB binding site in a conserved region close to the transcription start site of YAP gene (Fig. 1A). Consistent with prior studies 15, treatment with 5 μM RA for 24 hrs resulted in robust neurite outgrowth in N2a cells (Fig. 1B). This is accompanied by sustained Ser133 phosphorylation of CREB, while the abundance of total CREB was largely unaffected (Fig. 1C). Interestingly, treatment with RA also resulted in a potent increase in YAP expression. To examine whether induced expression of YAP was directly regulated by CREB, we performed ChIP assay using an antibody against CREB and a control IgG antibody. Our results showed that CREB was specifically cross‐linked with the YAP promoter region containing the CREB binding site, but not with YAP exon2 and intron2 regions, which were used as negative controls (Fig. 1D). Furthermore, incubation with a specific inhibitor of CREB phosphorylation, namely H89, could potently abolish RA‐induced YAP expression, and YAP protein expression was even lower than in cells without RA treatment. Together, these results support that YAP is a direct target of CREB in RA‐induced neurite outgrowth.

### YAP knockout impairs neurite outgrowth

**Figure 2:**
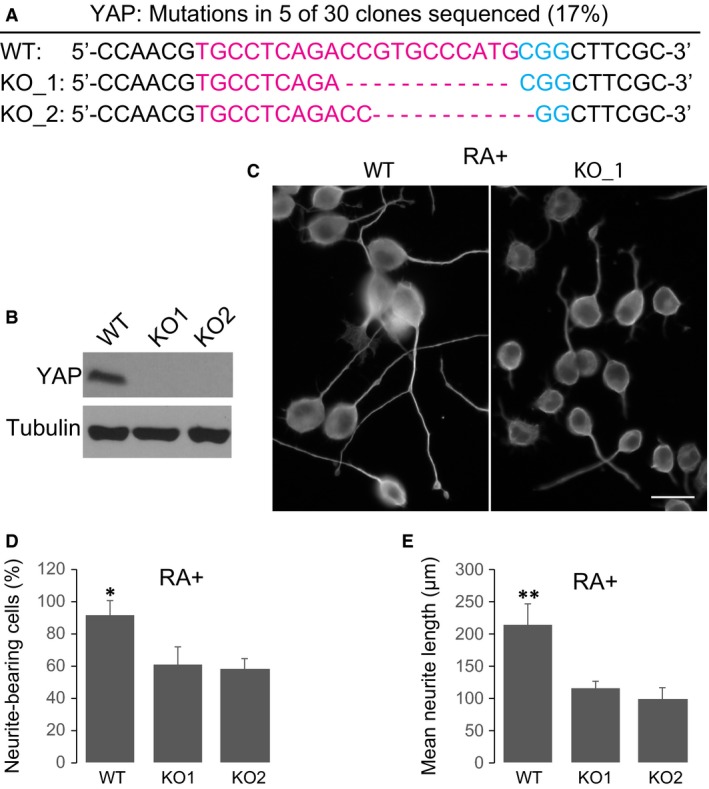
Yes‐associated protein (YAP) knockout impairs neurite outgrowth. (**A**) Alignment of Sanger sequencing results of PCR amplicons encompassing the target site of YAP by CRISPR/Cas. SgRNAs targets are highlighted in red, and the PAM sequence is blue. Dashes, nucleotide deletions. (**B**) Western blotting analysis of YAP expression in WT and two KO cells. (**C**) Representative data of RA‐induced neurite outgrowth in WT cells or YAP KO cells. (**D**) Quantification of the percentage of neurite‐bearing cells in WT and KO cells. (**E**) Quantification of neurite length in WT and KO cells. Data show the Mean ± S.E.M. values of three replicates (*P < 0.05, **P < 0.001).

To examine whether YAP plays any role in CREB‐mediated transcriptional responses governing neurite outgrowth, we established YAP knockout (KO) cell lines using the CRISPR/cas9 system. We designed a sgRNA targeting exon1 of YAP gene and identified mutations/deletions in five of 30 (17%) clones sequenced (Fig. 2A). Of them, two clones with frame‐shift mutation (KO_1 (‐11) and KO_2 (‐10)) showed complete depletion of YAP protein expression (Fig. 2B), probably *via* nonsense‐mediated degradation. As shown in Figure S1, YAP knockout resulted in a slight increase in cell death and a minor inhibition of cell proliferation. Interestingly, depletion of YAP in both KO cell lines leads to a significant decrease in the number of neurite‐bearing cells as compared with wild‐type (WT) cells upon RA treatment (Fig. 2C and D, *P* < 0.05). We also observed a dramatic reduction in neurite length in YAP KO cells bearing neurite (Fig. 2C and E, *P* < 0.001). This is in line with previous observation that up‐regulation of YAP *via* ROCK inhibition could significantly enhance neurite outgrowth in neural stem cells 11. These results indicate that YAP is a major mediator of CREB‐dependent neurite outgrowth activated by RA.

### YAP interacts with CREB *via* its N‐terminal domain

**Figure 3:**
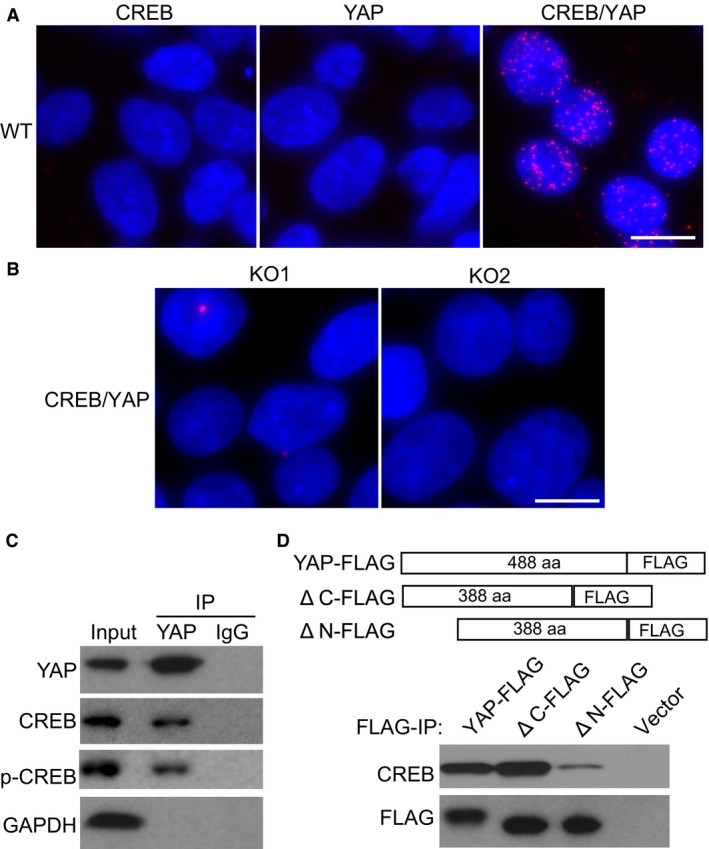
YAP interacts with CREB *via* its N‐terminal domain. (**A**) Proximity ligation assay of YAP and CREB interaction in wild‐type N2a cells treated with RA. Scale bars: 10 μm. (**B**) Proximity ligation assay of YAP and CREB interaction in YAP knockout cells treated with RA. Scale bars: 10 μm. (**C**) Immunoprecipitation of endogenous YAP and Western blotting probing YAP, p‐CREB, CREB and GAPDH. (**D**) Immunoprecipitation and Western blotting of FLAG‐tagged full‐length or C‐/N‐terminal deletions of YAP transiently expressed in N2a cells.

Previous studies suggested that CREB and YAP colocalized in liver cancer cells and co‐immunoprecipitated with one another 13. To investigate whether endogenous CREB interacts with YAP in N2a cells during neurite outgrowth, we performed a proximity ligation assay (PLA). This assay is a powerful tool to detect protein–protein interaction *in situ* by producing a strong PLA signal when two target proteins detected by antibodies are in close proximity (<40 nm). As shown in Figure 3A, CREB or YAP antibody alone produced no PLA signal, while combination of the two antibodies produced robust PLA signals exclusively observed in the nucleus region of N2a cells treated with RA. Moreover, no CREB/YAP PLA signal was detected in YAP knockout cells (Fig. 3B). This result provided direct evidence of interaction between endogenous CREB and YAP *in vivo*. To confirm the interaction between YAP and CREB, we performed YAP IP in RA‐treated N2a cell nuclear extracts and probed for YAP, CREB, phosphorylated CREB (p‐CREB) and GAPDH using Western blotting. As shown in Figure 3B, YAP was potently enriched in YAP IP, and the interactions between YAP and CREB as well as p‐CREB were observed. In contrast, no interaction between YAP and GAPDH was observed, while none of the proteins were detectable in IgG control IP. Notably, we did not observe any difference in YAP binding between total CREB and p‐CREB, suggesting that YAP binds both to phosphorylated and unphosphorylated version of CREB.

Prior structure analysis suggested that YAP contains a TEAD factor‐binding domain that is located at the N‐terminal domain and a transcriptional activation domain located at the C‐terminal of the protein 17. To investigate whether N‐terminal or C‐terminal domain was essential for YAP‐CREB interaction, we made full‐length YAP construct with FLAG tag and two mutant construct of similar size with N‐terminal or C‐terminal domain deletion (Fig. 3C). N2a cells were transfected with these constructs, and FLAG IP was performed. Interestingly, depletion of C‐terminal domain had no effect on YAP‐CREB interaction, while deletion of N‐terminal domain dramatically abolished YAP‐CREB interaction (Fig. 3C). These results established that YAP could interact with CREB *via* its N‐terminal domain.

### YAP knockout results in CREB degradation and reduced transcription activity

**Figure 4:**
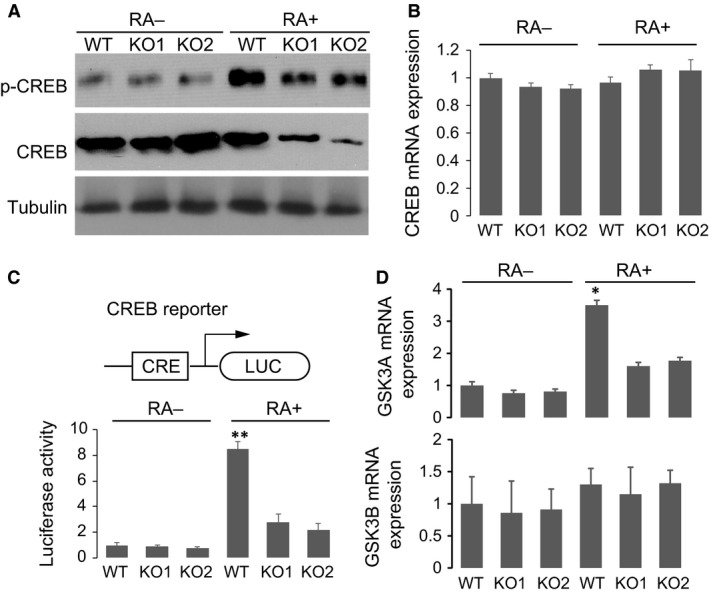
YAP knockout results in CREB degradation. (**A**) Western blotting of p‐CREB, CREB and tubulin in WT or YAP KO cell lines with or without RA treatment. (**B**) Real‐time PCR of CREB expression in WT or YAP KO cells. (**C**) Schematic of CREB transcription activity luciferase reporter. Luciferase activity of the reporter gene in WT and YAP KO cells with or without RA treatment. (**D**) Real‐time PCR assay of GSK3A and GSK3B expression in wild‐type and YAP knockout cells with or without RA treatment. Mean ± S.E.M. values of three replicates were shown (*P < 0.05, **P < 0.001).

We examined CREB and p‐CREB expression in WT and YAP KO cell lines. Interestingly, without RA treatment, p‐CREB level was low in all cell lines, and total CREB was not affected even when YAP was depleted (Fig. 4A). In striking contrast, however, upon RA treatment, both p‐CREB level and total CREB level were potently reduced in YAP KO cells, indicating that YAP is required to maintain the stability of phosphorylated form of CREB (Fig. 4A). Using real‐time PCR, we found that mRNA expression of CREB was unaffected in all cell lines upon RA treatment (Fig. 4B), confirming that the changes in CREB expression were occurred at protein level but not at transcription level. We further transfected these cell lines with a specific luciferase reporter for CREB activity and treated the cells with RA. The promoter of the reporter gene contains a strong CRE motif that binds CREB (Fig. 4C). As expected, RA treatment activated CREB and potently enhanced luciferase activity of the reporter gene in WT cells. In striking contrast, however, YAP depletion resulted in robust reduction in luciferase activity in KO cells as compared with WT cells. Furthermore, we used real‐time PCR to examine the expression of an endogenous CREB target GSK3A and a negative control GSK3B, which is not regulated by CREB 18. Our results showed that YAP KO significantly impaired RA‐induced GSK3A transcription, but had little effect on GSK3B expression. This is consistent with YAP being important in maintaining CREB stability in liver cancer cells 13 and might explain defects in neurite outgrowth observed in YAP KO cells (Fig. 2C–E).

### YAP stabilizes phosphorylated CREB upon RA treatment

**Figure 5:**
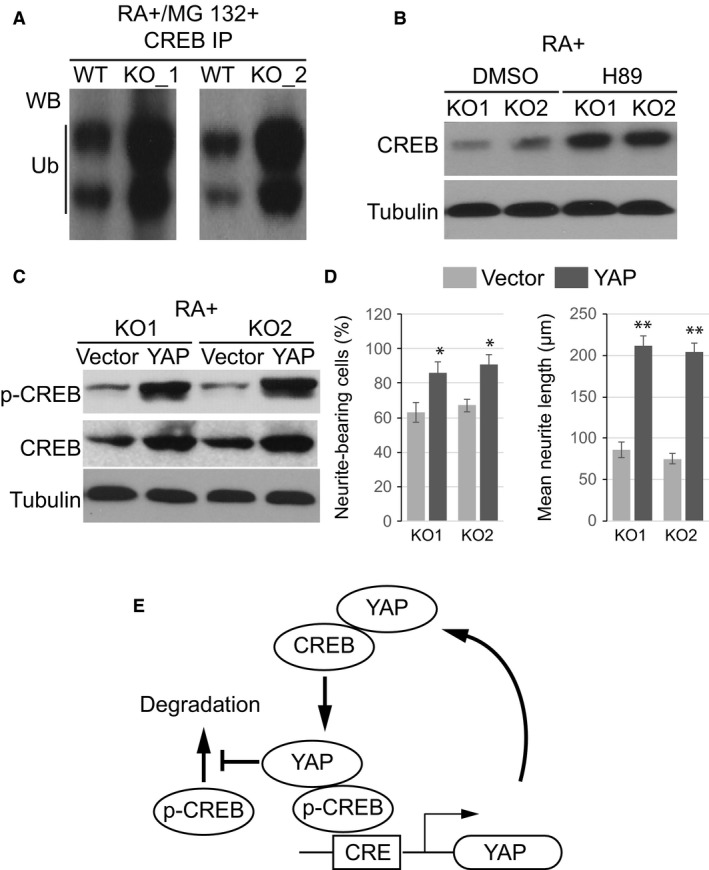
YAP stabilizes phosphorylated CREB upon RA treatment. (**A**) Endogenous CREB was immunoprecipitated from WT and YAP KO cells after treatment with MG132 to block degradation and probed for ubiquitination (Ub). (**B**) Western blotting of CREB in cells treated with RA and H89. (**C**) Western blotting of p‐CREB and CREB after transiently expressed YAP in knockout cells. (**D**) Quantification of the percentage of neurite‐bearing cells and average neurite length in KO cells after transfected with YAP or empty vector. Data show the Mean ± S.E.M. values of three replicates (*P < 0.05, **P < 0.001).

Previous studies suggested that YAP could prevent protein ubiquitin *via* inhibiting the function of E3 ubiquitin ligase 19. To address the relationship between YAP depletion and CREB instability upon RA treatment, we treated KO cells with proteasome inhibitor mg132 to allow accumulation of protein degradation intermediate. CREB was enriched by IP and probed with ubiquitin antibody. As shown in Figure 5A, YAP KO resulted in significant accumulation of ubiquitinated CREB, consistent with enhanced protein degradation activity. Furthermore, treatment with CREB phosphorylation inhibitor H89 largely abolished RA‐induced degradation of CREB in both KO cell lines (Fig. 5B), providing direct evidence that p‐CREB, but not total CREB, was unstable upon RA treatment when YAP was missing. Furthermore, transfection with full‐length YAP could efficiently stabilize CREB phosphorylation and largely restore CREB expression in YAP KO cells (Fig. 5C). Consistently, in both KO cell lines, transient expression of YAP could potently enhance the number of neurite‐bearing cells as well as restore neurite length upon RA induction (Fig. 5D). To further investigate whether the interaction between YAP and CREB is essential for maintaining CREB stability, we transfected YAP knockout cells with ΔN‐ or ΔC‐terminal YAP. Consistent with our findings that N‐terminal is essential for YAP‐CREB interaction, our results showed that ΔC‐terminal YAP, but not ΔN‐terminal YAP, could restore CREB expression and neurite outgrowth in these cells (Fig. S2 A and B). Together, these results revealed that CREB and YAP form a positive feedback loop to promote neurite outgrowth (Fig. 5E). In this model, activated by neurite outgrowth stimuli, CREB induces YAP expression, while YAP in turn binds to CREB and stabilizes phosphorylated CREB, preserving its transcriptional activity to mediate neuron differentiation.

## Discussion

Activation of the transcription factor CREB is a major mediator of neuronal differentiation in response to a diverse array of extracellular signals. Recent studies have identified a number of signalling cascades regulating the activity of CREB; however, little is known about the molecular mechanisms governing CREB stability and turnover after activation. In N2a cells, CREB activity is stimulated in response to RA and other agents that induce neuronal differentiation. Using this system, we identified YAP as a direct target of CREB during neurite outgrowth. Interestingly, our results showed that YAP directly interacts with CREB *via* its N‐terminal domain and plays a critical role in maintaining the stability of phosphorylated CREB. As CREB phosphorylation is critical for neurotrophic responses and CREB‐dependent transcription network 6, 20, we suggested that CREB and YAP form a positive feedback loop to stabilize CREB function and promote neurite outgrowth.

An interesting question is why YAP depletion causes degradation of p‐CREB upon RA treatment? Given the direct interaction between YAP and CREB, one possibility is that YAP functions as a cofactor of the CREB transcription complex, while loss of YAP results in abnormal assembly, dysfunction and finally degradation of the activated CREB complex. Moreover, it has been shown that YAP could directly abolish the function of E3 ubiquitin ligase *via* recruiting tyrosine kinase c‐Abl 19. Accordingly, another possible mechanism is that YAP protects CREB from ubiquitin ligase and degradation. This is demonstrated by our findings that significantly higher level of ubiquitinated CREB was observed in YAP knockout cells. Further studies are required to dissect the detailed molecular mechanisms underlying the relationship between YAP and CREB stability. As YAP might also function as a transcription regulator itself, it is still possible that some downstream target of YAP might be implicated in regulating CREB complex stability and function. Future transcriptome studies and ChIP studies will be needed to determine the co‐occupancies of YAP and CREB targets during neurite outgrowth.

As CREB‐mediated transcription is a convergence point for multiple signalling cascades and thus plays a variety of roles in diverse cell context 3, 14, 21, 22, regulation of CREB by YAP is not restricted to neurite outgrowth in N2a cells. For example, our results are consistent with previous findings that ROCK inhibition enhances neurite outgrowth by up‐regulating YAP expression in neural stem cells 11. As CREB activation has been strongly implicated in a wide range of neuronal differentiation and biogenesis processes, the regulatory feedback loop between CREB and YAP might be widespread in different cell types. Thus, the neuritogenic effects of CREB/YAP establish them as promising therapeutic targets for central nervous system injury and neurodegenerative disorders.

## Supporting information


**Figure S1** Cell death and proliferation of YAP knockout cells.

**Figure S2** N‐terminal YAP stabilizes phosphorylated CREB upon RA treatment.
